# Effectiveness of resveratrol as a root canal irrigation solution in inhibiting biofilm and bacterial metabolic activity of *Enterococcus faecalis*

**DOI:** 10.1007/s10103-026-04870-z

**Published:** 2026-04-10

**Authors:** Merve Uysal, Bulem Üreyen Kaya, Emel Sesli Çetin, Göksel Bilir

**Affiliations:** 1 Private Clinic, İzmir, Turkey; 2 Faculty of Dentistry, Department of Endodontics, Süleyman Demirel University, Isparta, Turkey; 3 Faculty of Medicine, Department of Microbiology, Süleyman Demirel University, Isparta, Turkey

**Keywords:** Antimicrobial, Biofilm, Diode laser, Metabolic activity, Resveratrol

## Abstract

Photodynamic therapy is a promising alternative to standard antimicrobial intracanal cleaning and shaping. The potential of resveratrol, a natural phytoalexin, as a photosensitizing and antibacterial agent for endodontic therapy remains uninvestigated. The aim of this study was to assess the effects of resveratrol, either alone as a root canal irrigation solution or activated with a diode laser, on *Enterococcus faecalis (E. faecalis*) biofilms, focusing on its antibiofilm and bacterial metabolic activities. Mandibular premolars were inoculated with *E. faecalis* for four weeks and subsequently disinfected using one of five methods (NaCl (0.9%); NaOCl (2.5%); resveratrol (128 µg/mL); resveratrol with diode laser (940 nm); diode laser). Microbial counts were measured before and after disinfection using colony-forming units. Dehydrogenase enzyme activity and polysaccharide synthesis of *E. faecalis* were also evaluated. Disinfection with the resveratrol with diode laser and NaOCl showed significantly greater bacterial reduction than the other disinfection protocols (*p*<0.001). No significant differences in dehydrogenase activities among the disinfection protocols (*p*>0.05). The lowest amount of polysaccharide was found in the resveratrol with diode laser group (*p*<0.001). The photoactivation of resveratrol with a diode laser may serve as an effective therapeutic strategy for reducing bacterial load and inhibiting polysaccharide formation.

## Introduction

The primary objective of endodontic treatment is to disinfect the root canals to reduce the bacterial load. This can be achieved through the use of irrigation solutions and antibacterial agents, as well as by mechanically shaping the root canal. Several studies have shown that, despite being a crucial component of root canal therapy, chemomechanical shaping has limitations since bacteria can penetrate deeply into complex anatomical structures, such as auxiliary canals, apical branches, isthmuses, and dentinal tubules [1].

Resveratrol (3,4’,5-trihydroxystilbene) is a natural phytoalexin found in many plants, particularly grapes, with osteogenic and anti-inflammatory effects of interest in dentistry [2]. Resveratrol decreases polysaccharide synthesis and associated virulence gene expression, reducing the virulence characteristics, acid production, and acid tolerance of *Streptococcus mutans* [3]. It has an antibacterial effect on *Porphyromonas gingivalis* by inhibiting bacterial adherence and supressing the inflammatory response [4]. It may also bind to Cu(II) to create a Cu(II)-peroxide complex, which can lead to DNA breakage and cell death [5]. Additionally, resveratrol’s reduction in cellular energy generation and inhibition of the electron transport chain (ETC) and F0F1-ATPase can suppress the proliferation of pathogenic microbes.

Resveratrol has been reported to have photodynamic activity by producing singlet oxygen after light stimulation, which increases the production of proinflammatory cytokines (TNF-α and IL-17), reduces inflammation, induces myeloperoxidase (MPO) and E-cadherin expression, and supports IL-10 production and bacterial inhibition [6]. Although resveratrol exhibits antimicrobial and photodynamic properties, its potential as a photosensitizer in endodontic photodynamic therapy has not been investigated. Therefore, this study evaluated whether diode-laser activation of resveratrol enhances its antibiofilm efficacy against *E. faecalis* biofilms within infected root canals.The hypothesis is that there are no statistically significant differences among the mean antibiofilm and metabolic inhibitory effects of resveratrol alone, light-activated resveratrol, and 2.5% NaOCl on *E. faecalis* biofilms.

## Methodology

The sample size was calculated using G*Power 3.1 software (Heinrich–Heine–Universität, Düsseldorf, Germany). Based on data from Ye et al. (2019) [7], with a significance level of 0.05, a power of 0.95, and an effect size of 8.06, the minimum requirement was calculated as two samples per group. To ensure statistical robustness, the final sample size was set at *n* = 10 per group. This study, approved by the Süleyman Demirel……. University Ethics Committee (no. 29.06.2021/225) and conducted according to the Declaration of Helsinki, version 2008. Following radiographic screening, 64 extracted human mandibular premolars were selected for a single, straight, and round canal. Exclusion criteria included caries, fractures, calcifications, complex canal anatomy, or prior endodontic treatment.

Crowns were sectioned to standardize root lengths to 14 mm. After confirming apical patency (size 10 K-file, Dentsply Sirona, Ballaigues, Switzerland), working length was established 1 mm from the foramen. For inclusion, canals were required to have an initial apical diameter of ≤ 15.

Root canals were prepared using the crown-down technique with 0.06 taper EndoArt Gold NiTi files (İnci Dental Tıbbi Malz. San. Ve Tic. Ltd. Şti., İstanbul, Türkiye) to a size 30 master apical rotary instrument. Irrigation with 2 mL of 2.5% NaOCl (Cerkamed, Stalowa Wola, Poland) was performed after each instrument change. To neutralize any residual NaOCl, the root canal was irrigated with 2.5 mL of 5% sodium thiosulfate (Na₂S₂O₃). The smear layer was then removed by irrigating with 5 mL of 17% EDTA for 1 min, followed by 5 mL of distilled water for another minute. The apical foramen of each root was sealed using nail varnish.

Roots were sterilized by autoclaving (121 °C, 15 psi, 40 min) while individually immersed in BHI broth (Merck KGaA, Darmstadt, Germany) in 1.5 mL Eppendorf tubes. A subsequent 48-hour incubation at 37 °C confirmed sterility.

### Contamination of the roots with *E. faecalis*

To create an *E. faecalis* (ATCC 29212) biofilm, 54 canals were inoculated with 10 µL of a bacterial suspension standardized to 1 × 10⁷ CFU mL^− 1^. The inoculum was delivered apically with a size 15 K-file. Samples were then incubated for four weeks at 35 ± 2 °C and 95% humidity, with BHI replenished every 48 h. Throughout the incubation, biofilm viability and purity were confirmed via medium turbidity, Gram staining, and colony morphology on blood agar. Ten additional roots were used as negative controls, receiving only sterile BHI broth.

Biofilm formation was confirmed on four representative samples. Two were analyzed by scanning electron microscopy (SEM) to verify surface colonization. Two additional roots were processed for histology (3-week 14% EDTA demineralization, embedding, sectioning); subsequent Gram staining and light microscopy confirmed bacterial penetration into dentinal tubules.

### Resveratrol solution preparation

The minimum inhibitory concentration (MIC) of resveratrol against *E. faecalis*, as reported in the literature [8], was evaluated, with concentrations ranging from 2 to 512 µg/mL. Resveratrol powder (R5010, Sigma-Aldrich, St. Louis, MO, USA) was dissolved in 10 mL of dimethyl sulfoxide (DMSO) (İsolab Laborgeräte GmbH, Eschau, Germany), and a stock solution of 1024 µg/mL was prepared using sterile distilled water as the solvent, resulting in a final DMSO concentration of less than 1% to minimize any potential impact on bacterial growth [9].

### Determination of minimum inhibitory concentration (MIC) and minimum bactericidal concentration (MBC)

Resveratrol MIC and MBC against *E. faecalis* were determined via a CLSI-standard broth microdilution assay. Serial dilutions in MHB were inoculated with *E. faecalis* (~ 5 × 10⁵ CFU mL^− 1^) and incubated (37 °C, 24 h). The MIC was the lowest concentration inhibiting growth, as visualized by resazurin (R7017; Sigma-Aldrich). The MBC was the lowest concentration yielding ≥ 99.9% bactericidal activity upon subculturing from non-turbid wells onto BHI agar. All experiments were performed in triplicate and independently repeated twice.

### Crystal violet assay

A crystal violet assay was used to quantify *E. faecalis* biofilm biomass following photodynamic therapy with resveratrol (128 µg/mL) activated by either a diode laser (EPIC X, Biolase Inc., Irvine, USA) with an endodontic tip (E2-14) or LED (Woodpecker LED C, Guilin Woodpecker Medical Instrument Co., Ltd., Guilin, China). Biofilms were grown for 48 h at 37 °C in 6-well plates containing a 1:1 mixture of BHI broth and a 1 McFarland *E. faecalis* suspension. Untreated biofilms served as positive controls, and wells with sterile medium served as negative controls. After incubation, non-adherent cells were removed by aspirating the supernatant and rinsing each well twice with 200 µL of sterile saline.

The established biofilms were randomly assigned to one of three experimental groups (*n* = 2 wells per group) as detailed in Table 1. In all groups, the photosensitizer (128 µg/mL resveratrol) was allowed a 5-minute pre-incubation period before light activation.

**Table 1:** Crystal violet assay experimental groups

Group	PS (Pre-incubation)	Light Activation Protocol
PS Only	128 µg/mL Resveratrol (5 min)	None
PDT-Laser	128 µg/mL Resveratrol (5 min)	Diode Laser (940 nm, 0.9 W, pulsed): 4 cycles of 5s on / 10s off
PDT-LED	128 µg/mL Resveratrol (5 min)	LED Light: 20s continuous exposure

*PS* photosensitizer

*PDT* photodynamic therapy

After treatment and two saline rinses, the remaining biofilm in each well was fixed with methanol (15 min), air-dried, and stained with 1% crystal violet (15 min). Excess stain was rinsed off, and the retained dye was solubilized in 200 µL of ethanol. Biofilm biomass was quantified by measuring the absorbance at 570 nm (OD₅₇₀) with a microplate spectrophotometer (Synergy™ HTX spectrophotometer; BioTek Instruments, Winooski, Vermont, USA). Results were calculated as the percentage of biomass reduction relative to the positive control. The experiment was performed in triplicate and repeated three times.

### *Ex vivo E. faecalis* biofilm assay

Following biofilm formation, samples were randomly allocated into five experimental groups (*n* = 10 per group). For all groups, a total of 5 mL of irrigant was delivered using a 30-G side-vented needle placed 1 mm from the working length. The specific chemo-mechanical protocols for each group are detailed in Table 2.

**Table 2:** *Ex Vivo E. faecalis* biofilm assay experimental groups

Group	Primary Irrigant	Light Activation Protocol
Negative Control	5 mL of 0.9% NaCl	None
Positive Control	5 mL of 2.5% NaOCl	None
PS Only	5 mL of 128 µg/mL Resveratrol	None
PDT	5 mL of 128 µg/mL Resveratrol	Diode Laser (940 nm, 0.9 W, pulsed (repetition rate up to 20kHz)): 5-min pre-incubation. 4 cycles of 5s activation / 10s rest. Irrigant refreshed during rests. E2-14 endodontic tip moved apico-coronally
Laser Only	5 mL of 0.9% NaCl	Diode Laser (940 nm, 0.9 W, pulsed): Protocol identical to Group PDT

*PS* photosensitizer

*PDT* photodynamic therapy

Bacterial loads were assessed before (S1) and after (S2) disinfection. Following a saline rinse to remove planktonic cells, each canal was sampled using three sequential size 30 paper points (1 min each). The collected paper points were vortexed in 1 mL of saline to create a bacterial suspension. This suspension was then serially diluted, and 100 µL aliquots were plated in triplicate on BHI agar. After a 24-hour incubation at 35 °C, colony-forming units (CFUs) were counted. Treatment efficacy was evaluated by calculating both the absolute CFU mL^− 1^ and the percentage reduction from S1 to S2.

### Confocal laser scanning microscope (CLSM)

One specimen from each group was split into two halves (mesial and distal) using a chisel and mallet. Each half was stained using the LIVE/DEAD BacLight™ Bacterial Viability Kit (L10316; Filmtracer™ Invitrogen™, Thermo Fisher Scientific, Massachusetts, USA), following the manufacturer’s instructions. The specimens were examined by CLSM (LSM 800, Carl Zeiss Microscopy GmbH, Jena, Germany) using 40 x oil lens and immersion oil. For each specimen, two randomly selected areas (159.73 μm x 159.73 μm each) were scanned.

### XTT assay

The metabolic activity of surviving *E. faecalis* in the S2 samples was quantified using an XTT assay (Cayman Chemical, Ann Arbor, Michigan, USA). Briefly, S2 samples were mixed 1:1 with fresh BHI broth and incubated for 48 h at 37 °C to allow for bacterial recovery. Aliquots (100 µL) of the recovered culture were then mixed with the XTT reagent in a microplate, incubated (4 h, 37 °C, dark), and the resulting formazan production was measured spectrophotometrically at 492 nm (reference 690 nm). Sterile medium was used for blank correction.

### Polysaccharide synthesis

The production of water-insoluble polysaccharides by surviving bacteria in S2 samples was quantified using the phenol-sulfuric acid method [10]. Following a 48-hour biofilm regrowth period in BHI, the samples were pelleted by centrifugation (4000 rpm, 6 min). The pellet was then resuspended in deionized water and reacted with 5% phenol and concentrated sulfuric acid. After a 30-minute incubation, the absorbance of the solution was measured at 490 nm. Final polysaccharide concentrations (mg/L) were determined by referencing a glucose standard curve [11].

### Statistical analyses

Following a Shapiro-Wilk test, non-parametric analyses were required for the biofilm reduction (log₁₀-transformed CFU) and XTT metabolic activity data; these were compared using the Kruskal-Wallis test with Dunn’s post hoc test. Normally distributed polysaccharide data were analyzed by factorial measures ANOVA with Tukey’s post hoc test. Significance was set at *p* < 0.05 for all analyses.

## Results

**Fig. 1:**
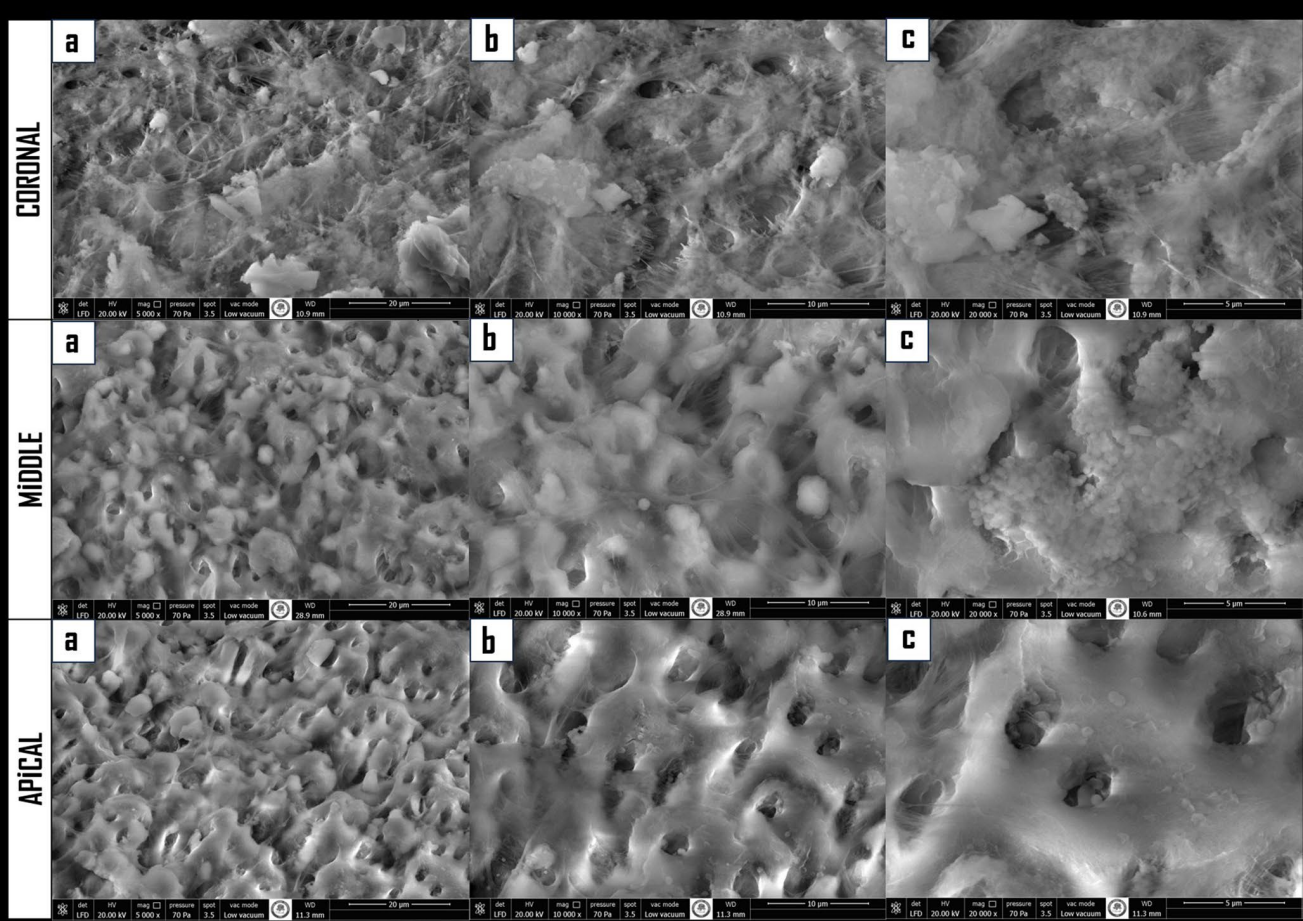
Infected root canals. Bioﬁlm-like structures were observed on the surface of the root canal walls at (**a**) 5000 (**b**)10 000 (**c**) 20 000 magniﬁcations

 SEM images revealed that biofilm-like structures were observed on the surface of the root canal walls (shown in Fig. 1), and the penetration of bacteria into the dentinal tubules was confirmed by light microscopy.

### Determination of MIC and MBC

The MIC and MBC values of resveratrol against *E. faecalis* ATCC 29212 were 128 µg/mL and 1024 µg/mL, respectively.

### Crystal violet assay

 A reduction in *E. faecalis* biofilm biomass was observed for all treatments when compared to the positive control, with the results detailed in Table 3. Resveratrol with diode laser (57.04%) resulted in greater *E. faecalis* biofilm biomass reduction than resveratrol with LED (35.65%) or resveratrol alone (11.56%).

**Table 3:** Quantification of *E. faecalis* biofilm biomass following treatment, measured by crystal violet absorbance at 570 nm

	Treatment absorbance	Control absorbance	Biofilm reduction (%)
Resveratrol	0.757± 0.012	0.856± 0.074	11.56
Resveratrol with diode laser	0.439± 0.021	1.022± 0.065	57.04
Resveratrol with LED	0.591± 0.024	0.92± 0.13	35.65

### *Ex vivo E. faecalis* biofilm assay

 No bacterial growth was observed in the negative controls. The samples taken before disinfection (S0) had bacterial load of approximately 6.298 log CFU. Table 4 presents the mean, median, and range differences between the bacteriological samples taken before and after the disinfection protocols. Disinfection with resveratrol with diode laser (median reduction 6.00 log_10_ CFU mL^− 1^) and NaOCl (median reduction 5.00 log_10_ CFU mL^− 1^) resulted in significantly greater bacterial reduction than the other disinfection protocols (*p* < 0.001). Although bacterial counts were reduced after the disinfection protocols, all specimens still showed bacterial growth.

**Table 4:** Reduction of bacterial load after disinfection protocols (mean, standard deviation, median of log10 CFU mL^−1^ and percentage reduction)

Disinfection Protocols	*n*	mean	SD	Median	Rank	%
NaCl	10	1.14	0.95	1.00	12.6^C^	17.68
NaOCl	10	4.76	1.77	5.00	36.6^A^	79.23
Resveratrol	10	2.84	1.39	2.65	25.9^B^	48.78
Resveratrol with diode laser	10	5.28	1.85	6.00	39.6^A^	77.89
Diode laser	10	1.18	0.64	1.00	12.9^C^	18.38

Different letters indicate significant differences ( *p* < 0.001)

### CLSM

**Fig. 2:**
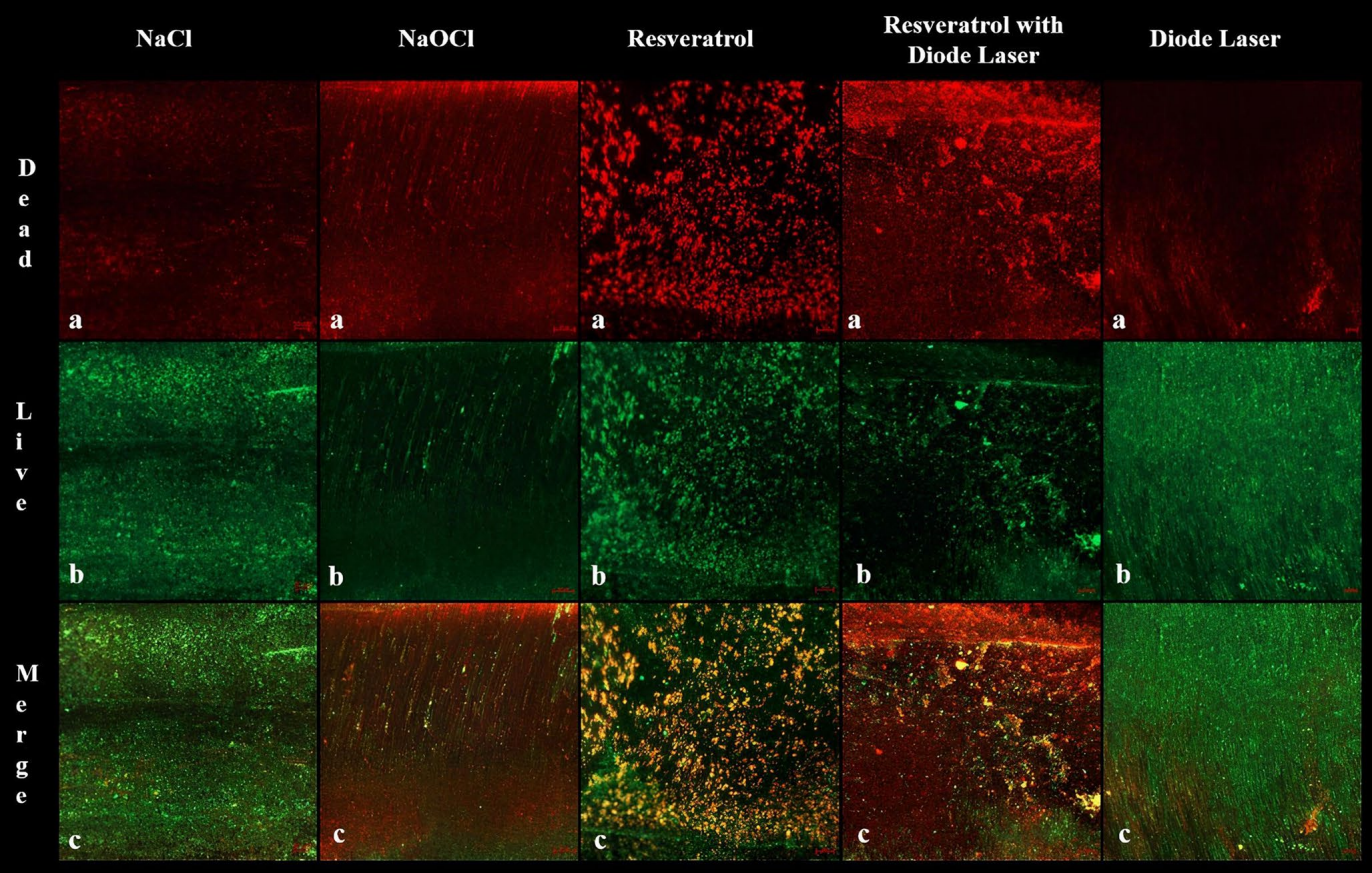
CLSM representative images of *E. faecalis* biofilms after disinfection protocols, green fluorescence indicates live bacteria while red indicates dead bacteria

 The qualitative analysis of the CLSM images revealed that specimens treated with NaOCl and resveratrol with diode laser exhibited more intense red fluorescence than the other specimens. Green fluorescence indicates live bacteria while red indicates dead bacteria. CLSM representative images of *E. faecalis* biofilms after disinfection protocols are shown in Fig. 2.

### XTT assay

 Due to the absence of formazan formation in two samples per group, these specimens were excluded from the analysis. Consequently, the final sample size for the XTT assay was 40 (*n* = 8 per group). Results of the XTT assays are shown in Table 5. There was no significant difference in dehydrogenase activities among the disinfection protocols (*p* = 0. 092).

**Table 5:** Dehydrogenase activities (absorbance values) of the disinfection protocols derived from XTT assay (*p*> 0.05)

Disinfection Protocols	*n*	mean	SD	Median	Min-max
NaCl	8	1.29	0.06	1.27	1.20-1.37
NaOCl	8	0.96	0.35	0.93	0.35-1.36
Resveratrol	8	1.22	0.38	1.29	0.61-1.66
Resveratrol with diode laser	8	1.03	0.52	0.96	0.43-0.71
Diode laser	8	1.4	0.31	1.48	0.65-1.58

### Polysaccharide synthesis

 One specimen per group was lost during the experimental procedures, except within the NaOCl positive control group. Polysaccharide production by *E. faecalis* is shown in Table 6. Significant differences were observed among the disinfection protocols for metabolic product (*p* < 0.001). The highest amount of polysaccharide was in the saline group (1178.0 ± 239.8), and the lowest in the resveratrol with diode laser group (552.2 ± 152.3) (*p* < 0.001).

**Table 6:** Polysaccharide production (mg/L) by E. faecalis after disinfection protocols

Disinfection Protocols	*n*	mean± SD	Min-max
NaCl	9	1178.0 ±239.8^A^	914.94-1606.5
NaOCl	10	1052.2 ±281.9^A^	673.34-1381.64
Resveratrol	9	856 ±313^A, B^	392.26-1280.46
Resveratrol with diode laser	9	552.2 ±152.3^B^	229.24-707.07
Diode laser	9	1151 ±335^A^	701.44-1775.15

Different letters indicate significant differences (*p*<0.001)

## Discussion

Conventional endodontic treatment involves mechanical debridement combined with chemical irrigations. However, because of the anatomical complexity of the root canal system, eliminating residual bacteria is almost impossible. Increasing the concentration and duration of NaOCl application enhances its disinfecting efficacy; however, this also leads to higher tissue breakdown in dentin, deproteinization of dentin collagen, and cytotoxic effects in periapical tissues [12–14].

New methods have been proposed to enhance root canal disinfection, and photodynamic therapy (PDT) emerges as a promising alternative to traditional antimicrobial intracanal cleaning and shaping techniques. Studies have demonstrated that endodontic bacteria, including *E. faecalis*, are significantly reduced following root canal treatment with PDT compared to conventional treatment alone [15–17]. While high-efficiency lasers are often used as light sources in PDT due to the monochromatic light that can be directed through fiber optic tips, diode lasers are particularly preferred for their ease of use [18]. However, few studies compare LED light and diode lasers in terms of PDT efficiency. Asnaashari et al. (2016) showed that LED light at a wavelength of 630 nm was more effective than a diode laser at 810 nm in reducing *E. faecalis* in root canals [19]. On the other hand, diode laser activation (980 nm, power 0.5 W, with pulse settings) for a herbal extract showed better bacterial inactivation performance than that of blue LED light [20]. Resveratrol has an absorption band at 306 nm in the UV/visible light spectrum, with light emission close to its maximum absorption range. However, some studies have used photosensitizing agents activated at wavelengths outside the maximum absorption range [21, 22]. In our study, activating resveratrol with a diode laser at a wavelength of 940 nm before application to root canals exhibited more antibiofilm activity than LED light, as shown by the crystal violet test.

The results of this study revealed that there was a difference in terms of antibiofilm and metabolic inhibitory effects of resveratrol alone, light-activated resveratrol, and 2.5% NaOCl on *E. faecalis* biofilms and the null hypothesis was therefore rejected.

Using various photosensitizing agents with lasers for disinfection of infected root canals is important. This study is the first to report the use of resveratrol alone or in combination with a light source for root canal disinfection. No statistically significant difference was found between the use of a diode laser alone and the negative control group, consistent with the literature. De Meyer et al. (2017) showed that 2940 nm laser treatment with saline reduced bacterial counts by about 1 log_10_, whereas laser treatment with NaOCl reduced bacterial levels by > 2.2 log_10_ [23]. Similarly, Kreisler et al. (2003) reported that using an 809 nm diode laser alone was not more effective than the simultaneous use of the laser with NaOCl in vitro [24]. Using a 980 nm laser alone in a dry root canal is also significantly less effective than conventional chemomechanical therapy [25]. These findings suggest that while a diode laser cannot replace conventional treatments, it should be considered an adjunctive therapy [25].

The operational parameters of a 970-nm diode laser are critical determinants of its photothermal efficacy against *Enterococcus faecalis* biofilms. While previous research [26] has demonstrated that a 2 W continuous wave setting effectively reduces biofilm viability without inducing root dentin carbonization, the photothermal impact in the present study was likely negligible. This lack of thermal influence is attributed to the low power output (0.9 W), the pulsed emission mode, the brief 20-second irradiation interval, and the intermittent refreshing of the irrigant. Furthermore, the fiberoptic tip was utilized in a non-contact, helicoidal motion—transitioning from the apical to the coronal region—to further mitigate heat accumulation. The *ex vivo E. faecalis* biofilm assay revealed that diode laser irradiation alone yielded results comparable to the 0.9% NaCl control group. This outcome suggests that the laser parameters employed herein did not elicit a significant thermal effect, but rather functioned through photoactivation when used in synergy with a photosensitizer (PS), specifically resveratrol.

The resveratrol with a diode laser and NaOCl achieved a superior reduction in bacterial levels compared to other disinfection methods examined. When used independently, resveratrol displayed minimal antibacterial activity against *E. faecalis*, proving significantly less effective than its combination with a diode laser. As corroborated by other research [26], the diode laser amplifies the efficacy of the natural extracts. Representative CLSM imaging of randomly selected samples from each disinfection protocol further confirmed these results. Interpretation of the CLSM data was restricted to qualitative observation; the absence of quantitative analysis represents a limitation of the study.Although multispecies biofilms offer a closer resemblance to clinical reality, to ensure high reproducibility and minimize confounding variables associated with interspecies interactions, a monospecies biofilm model was utilized in this study. Notably, no treatment regimen fully eradicated bacteria from all samples. This highlights the inherent difficulty of endodontic disinfection, which can be attributed to the protective nature of microbial biofilms, the deep penetration of bacteria into dentinal tubules and complex anatomy of root canal system.

XTT tests, which assess bacterial cell metabolic activity, are often used as an additional method in antibiofilm evaluation studies [27]. The XTT assay’s principle is based on the reduction of the tetrazolium compound XTT to orange formazan by mitochondrial dehydrogenase enzymes in viable cells. Due to metabolic differences between microbial species and strains, XTT reduction and color changes may vary. Thus, using single-species biofilms in antibiofilm evaluations, as in our study, improves the accuracy of XTT findings [28].

Kuhn et al. (2003) noted that there is not always a linear relationship between the number of organisms and the colorimetric signal in tetrazolium assays, and different strains metabolize substrates with varying efficiencies. Therefore, interspecies comparisons require detailed standardization. They also noted that while the XTT formazan product is visible in solution, some strains may retain a significant amount of the product intracellularly, which becomes soluble only after cell treatment with DMSO. This retention can vary between different cellular states, such as planktonic and biofilm [28].

Zeng et al. (2021) evaluated the effectiveness of different irrigation activation methods in removing 21-day-old *E. faecalis* biofilm using culture methods, MTT, and XTT tests [29]. Both cell viability (MTT) and metabolic activity (XTT) were found to be high in the group with the highest CFU count by the culture method. This suggests that MTT and XTT tests can complement antibiofilm evaluations [29]. In this study, according to the XTT test results, the lowest bacterial viability was observed in the NaOCl and resveratrol/diode laser groups. Although this supports the CFU counts, no statistically significant differences were found among the groups regarding bacterial cell viability. The XTT test in this study was conducted after incubating bacterial samples from root canals in a medium for 48 h, which allowed sufficient time for bacterial samples to regrow. Even if the samples cannot form colonies in culture methods, bacteria in the VBNC (viable but non-culturable) phase can reproduce and show metabolic activity when conditions are restored, such as access to nutrients.

Polysaccharide production is a key virulence factor of cariogenic biofilms, including intracellular polysaccharides and extracellular polysaccharides (EPS) [30]. Lower polysaccharide amounts indicate easier exposure to antimicrobial agents [31]. Contrary to the results of the XTT test, a significant difference in polysaccharide levels was observed after the irrigation protocols. The polysaccharide amount was significantly lower in the group treated with resveratrol alone. Li et al. (2020) demonstrated the inhibitory effect of resveratrol on biofilm formation and polysaccharide synthesis in *Streptococcus* mutans [3]. Our study also supports that resveratrol inhibits polysaccharide formation, although its antibacterial activity is limited.

Despite the significant antibacterial reduction observed, several limitations must be acknowledged. The use of a monospecies biofilm in an *ex vivo *model simplifies the complex clinical environment, where polymicrobial synergy and host physiological factors play a critical role. Additionally, the lack of data regarding dentin penetration and cytotoxicity means that while the protocol is effective on the canal surface, its deep-tissue efficacy and biological safety profile require further validation before clinical implementation.

## Conclusions

Diode-laser activation of resveratrol significantly enhanced antibiofilm activity against *E. faecalis* biofilms in an *ex vivo* root canal model. However, bacterial eradication was not achieved, and further studies evaluating multispecies biofilms and clinical applicability are required.

## Data Availability

All data used and/or analysed during the current study are included in this published article.
